# Irritated and Feeling Better? Aging Parents’ Marital Status and Daily Interaction With Grown Children

**DOI:** 10.1093/geroni/igaa057.2188

**Published:** 2020-12-16

**Authors:** Yijung Kim, Kyungmin Kim, Karen Fingerman

**Affiliations:** 1 University of Massachusetts Boston, Boston, Massachusetts, United States; 2 The University of Texas at Austin, Austin, Texas, United States

## Abstract

Aging parents’ marital status shapes their ties to family members, but less is known about its link to their daily mood and interaction with grown children. This study examined married, widowed, or divorced/separated aging parents (N = 203, Mage = 79.80) from the Family Exchanges Study, who completed a 7-day daily diary on their daily mood (positive, negative) and interactions (any contact, irritable, enjoyable interaction) with the grown children (N = 771, Mage = 53.20). Findings from multilevel models indicated that widowed parents were more likely to report irritable interactions with their grown children than the married ones. Furthermore, married and widowed parents tended to report more negative mood, whereas separated parents tended to report less negative mood on days they had irritable interactions with grown children. This study highlights the centrality of aging parents’ daily interaction with grown children and suggests that the dynamics of family composition warrant attention.

